# Moeller’s Surgery

**Published:** 1892-04

**Authors:** 


### MOELLER'S SURGERY.

*Specielle Chirurgie fur Tierarzte, by Prof. Möller.*

Since the publication of Peuch and Toussaint, Precis de chirurgie vètèrinaire, in 1876, no good work on veterinary surgery has appeared in any language. A number of works have been issued, to be sure, some in part, some in full, but not one has filled the aching void in our professional literature. This fact does not, however, indicate that progress in veterinary surgery had come to a standstill; far from it. The periodicals and journals abound in records of discoveries and improvements and modifications of methods and reports of cases; all of which show that the veterinary surgeons were active and progressing, if they were not writing books.

This great need has, at last, been seen and supplied by Prof. Möller, of Berlin, whose *Specielle Chirurgie fur Thierarzte* appeared a few months ago.

Prof. Möller is a man of the greatest experience; he has for years had charge of the largest veterinary surgical clinic in the world. The surgical division of the Berlin hospital accommodates some forty to fifty horses, and it is constantly necessary to turn numbers away for lack of room, so that only the most interesting cases are retained. Prof. Möller is also a man of great energy and originality, and this is shown on every page of his book. He has not relied solely on his own experience, but has consulted everything in the German and foreign literature bearing on the subjects of which his book treats.

The description of the operation for rsaring will be read with great interest, as the author is really the originator of the method used at the present day. The chapter on diseases of the teeth contains the most exhaustive consideration of this subject that has yet appeared. The discussion of spavin is most thorough and original, and the treatment for which the best results are claimed is a subcutaneous periosteotomy.

The portions relating to symptomatology and diagnosis are exceedingly clear and interesting. The author has been able to surpass all of his predecessors in simplyfying the intricate subject of lameness, and was the first to make a careful study of the movements of locomotion and to tabulate them in what he calls "the analysis of the movements."

The parts relating to surgical pathology are rather incomplete, and this is the only shortcoming of the book. But perhaps it is as well not to enter into a field so imperfectly explored as that of veterinary surgical pathology, and to allow the students to consult Billroth and other standard works on the subject, rather than to apply, without modification the facts demonstrated in regard to man to the diseases of the domesticated animals; as was done by the author of a recent veterinary surgery (Hoffman). In shortening the discussion of these imperfectly understood subjects, Möller has shown his regard for scientific accuracy.

The book is nicely printed, on good paper, and contains a large number of illustrations, most of which are original.—PEARSON.

